# Intracellular S1P Generation Is Essential for S1P-Induced Motility of Human Lung Endothelial Cells: Role of Sphingosine Kinase 1 and S1P Lyase

**DOI:** 10.1371/journal.pone.0016571

**Published:** 2011-01-31

**Authors:** Evgeny V. Berdyshev, Irina Gorshkova, Peter Usatyuk, Satish Kalari, Yutong Zhao, Nigel J. Pyne, Susan Pyne, Roger A. Sabbadini, Joe G. N. Garcia, Viswanathan Natarajan

**Affiliations:** 1 Department of Medicine, The University of Illinois at Chicago, Chicago, Illinois, United States of America; 2 Department of Pharmacology, The University of Illinois at Chicago, Chicago, Illinois, United States of America; 3 Department of Cancer Biology, Beckman Research Institute, Duarte, California, United States of America; 4 Department of Medicine, University of Pittsburgh Medical Center, Pittsburg, Pennsylvania, United States of America; 5 Cell Biology Group, University of Strathclyde, Glasgow, United Kingdom; 6 Department of Biology, San Diego State University, and Lpath Inc., San Diego, California, United States of America; 7 Institute for Personalized Respiratory Medicine, The University of Illinois at Chicago, Chicago, Illinois, United States of America; Biological Research Center of the Hungarian Academy of Sciences, Hungary

## Abstract

**Background:**

Earlier we have shown that extracellular sphingosine-1-phosphate (S1P) induces migration of human pulmonary artery endothelial cells (HPAECs) through the activation of S1P_1_ receptor, PKCε, and PLD2-PKCζ-Rac1 signaling cascade. As endothelial cells generate intracellular S1P, here we have investigated the role of sphingosine kinases (SphKs) and S1P lyase (S1PL), that regulate intracellular S1P accumulation, in HPAEC motility.

**Methodology/Principal Findings:**

Inhibition of SphK activity with a SphK inhibitor 2-(p-Hydroxyanilino)-4-(p-Chlorophenyl) Thiazole or down-regulation of Sphk1, but not SphK2, with siRNA decreased S1P_int_, and attenuated S1P_ext_ or serum-induced motility of HPAECs. On the contrary, inhibition of S1PL with 4-deoxypyridoxine or knockdown of S1PL with siRNA increased S1P_int_ and potentiated motility of HPAECs to S1P_ext_ or serum. S1P_ext_ mediates cell motility through activation of Rac1 and IQGAP1 signal transduction in HPAECs. Silencing of SphK1 by siRNA attenuated Rac1 and IQGAP1 translocation to the cell periphery; however, knockdown of S1PL with siRNA or 4-deoxypyridoxine augmented activated Rac1 and stimulated Rac1 and IQGAP1 translocation to cell periphery. The increased cell motility mediated by down-regulation was S1PL was pertussis toxin sensitive suggesting “inside-out” signaling of intracellularly generated S1P. Although S1P did not accumulate significantly in media under basal or S1PL knockdown conditions, addition of sodium vanadate increased S1P levels in the medium and inside the cells most likely by blocking phosphatases including lipid phosphate phosphatases (LPPs). Furthermore, addition of anti-S1P mAb to the incubation medium blocked S1P_ext_ or 4-deoxypyridoxine-dependent endothelial cell motility.

**Conclusions/Significance:**

These results suggest S1P_ext_ mediated endothelial cell motility is dependent on intracellular S1P production, which is regulated, in part, by SphK1 and S1PL.

## Introduction

Sphingolipid metabolites such as ceramides and sphingoid bases are important modulators of cell survival, cell proliferation, angiogenesis, and vascular integrity. Among the various sphingolipids, sphingosine-1-phosphate (S1P), elicits a plethora of cellular responses such as proliferation, survival, chemotaxis and endothelial barrier regulation. S1P is a naturally occurring bioactive lipid found in nanomolar to micromolar concentrations in plasma and serum [1], and exerts its cellular responses through ligation to G-protein coupled S1P receptors, S1P_1–5_ that have been identified [2]. S1P receptors (S1PR) are differentially expressed in various cell types and are coupled to three distinct G-protein subfamilies, including G_i_, G_q_ and G_12/13_. S1PR activation results in down-stream activation of Rho-GTPases, cytoskeletal reorganization, adherens and tight junction assembly, and focal adhesion formation [3]–[6]. It is well established that S1P is a potent angiogenic and vascular maturation factor regulating endothelial cell proliferation, migration and remodeling [7]–[9]. Several signaling pathways including changes in [Ca^2+^]_i_, activation of phosphatidylinositol 3-kinase, Akt, MAPKs, Rac1 and PKC have been implicated in S1P-induced EC migration [2], [10], [11]. We have recently shown that S1P signals through S1P_1_ and G_i_ to activate PKC-ε and subsequently, a PLD2-PKC-ζ-Rac1 cascade to induce migration of human lung ECs [12]. These studies strongly suggest a role for extracellular action of S1P through S1P_1_ and other S1P-Rs in stimulating migration of ECs.

In addition to S1P's extracellular action, there is evidence that supports an intracellular role of S1P in calcium release [13], [14] and modulation of histone acetylation via HDACs in breast cancer cells [15]. Cellular S1P levels are regulated by its synthesis and catabolism. Sphingosine kinases (SphKs) 1 and 2 catalyze the phosphorylation of sphingosine (Sph) to S1P [16]–[18] while S1P is degraded back to Sph by S1P phosphatases 1 and 2 and lipid phosphate phosphatases [19]–[21] or to hexadecenal and ethanolamine phosphate by S1P lyase (S1PL) [22]–[25]. Availability of Sph is the rate limiting step in intracellular generation of S1P, and Sph is derived either from ceramides through ceramidases or from circulating plasma S1P through ecto-LPPs [21], [26]. Recent studies show that human lung ECs have the ability to utilize exogenously added S1P to generate intracellular S1P by hydrolysis to Sph catalyzed by LPPs and subsequent phosphorylation by SphKs [19]. In addition to these two pathways, S1P can also be generated in plasma by lysophospholipase D/autotaxin-mediated hydrolysis of sphingosylphosphorylcholine [27]; however, it is unclear if this pathway is a major source of plasma S1P.

The role of intracellular S1P or enzymes regulating the generation of cellular S1P in modulating cellular responses such as motility and proliferation is yet to be well established. Very little is known on intracellular targets of S1P and recent reports indicate potential interaction between S1P and histone deacetylase 2 in breast cancer cells [15] and S1P as a missing co-factor for E3 ubiquitin ligase TRAF2 in HEK 293 cells [28]. Further, part of the intracellularly generated S1P could be released by an ATP-binding cassette transporter, ABCC1, which may subsequently activate S1PRs in an autocrine or paracrine manner [29]. While platelets [30] and mast cells [29] have been shown to release S1P upon activation, human lung ECs released very little S1P under basal condition [31]. However, laminar shear stress leads to an increase in S1P release from ECs suggesting vascular endothelium as an important source of circulating S1P [32]. Interestingly, over-expression of SphK1 and SphK2 enhanced accumulation of intracellular S1P while knockdown of SphK1, but not SphK2, by siRNA decreased intracellular S1P production from extracellular S1P [19]. Conversely, down-regulation of S1PL with siRNA increased intracellular generation of S1P in the presence or absence of extracellular S1P in HPAECs [19]. As over-expression of SphKs or down-regulation of S1PL modulated S1P-induced human lung EC migration suggesting a potential role of SphKs and S1PL in cell motility, in the present study, we examined the role of SphKs and S1PL in human lung EC motility. Down-regulation of SphK1, but not SphK2, by siRNA as well as over-expression of SphK1 mutant attenuated intracellular S1P levels and serum- and S1P-induced migration of human pulmonary artery endothelial cells (HPAECs). However, knockdown of S1PL with siRNA enhanced intracellular S1P and the serum- and S1P-induced motility. Further, down-regulation of S1PL stimulated redistribution of Rac1 and IQGAP1 to cell periphery compared to scrambled siRNA transfected cells suggesting a potential role of intracellular S1P in regulating Rac1/IQGAP1 signaling and migration.

## Results

### Involvement of SphK1, but not SphK2, in Extracellular S1P-induced Wound Healing

**Figure 1:**
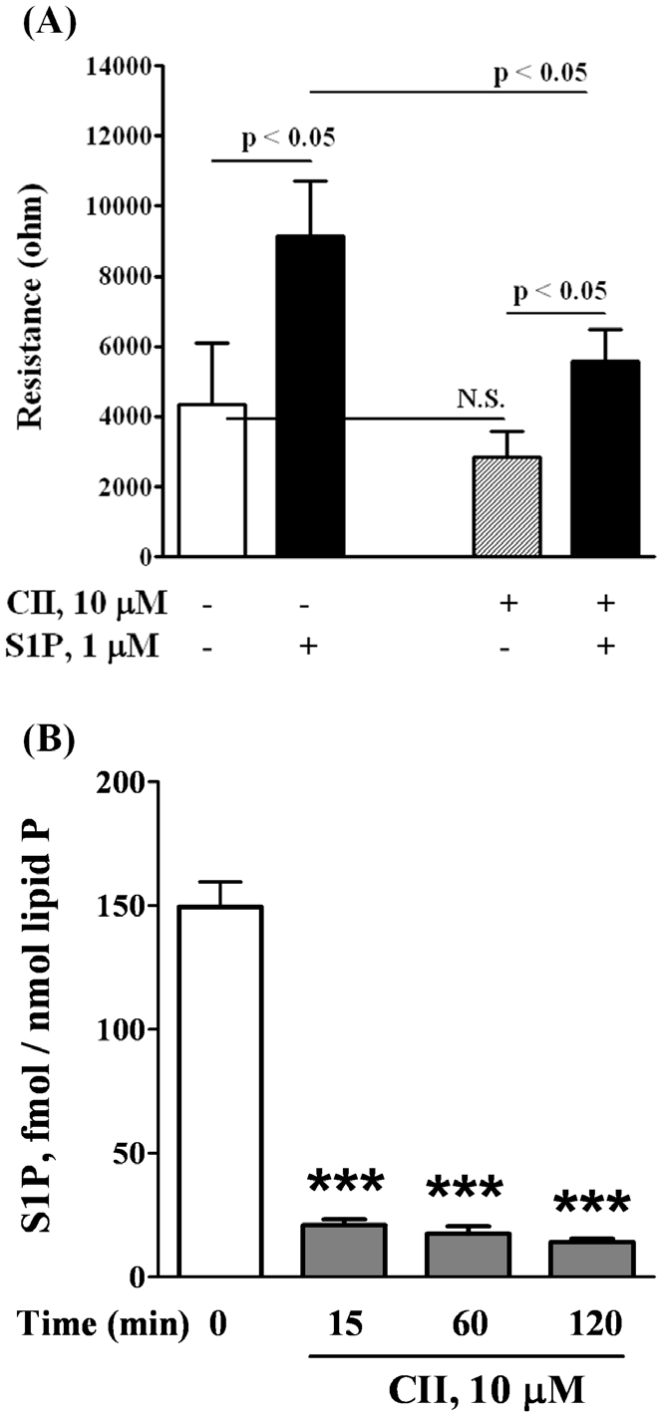
CII, inhibitor of SphK, decreases both the migration of HPAECs in a scratch assay *in vitro* and the content of S1P in HPAECs. HPAECs grown to ∼95% confluence in 35 mm dishes were starved for 3 h in 0.1% FBS in EBM-2 without growth factors and treated with 10 µM of CII. Monolayers were scratched, and challenged with medium containing 0.1% BSA or 1.0 µM S1P complexed to 0.1% BSA. ***A*** shows the migration of cells into a “wound” that was scratched and exposed to S1P. ***B*** shows the decrease of S1P content in cells as measured by LC/MS/MS (see Methods) after lipid extraction from harvested cells. The values are mean ± S.E.M for three independent experiments each performed in triplicate (*** - p<0.001 in comparison to T = 0 min).

**Figure 2:**
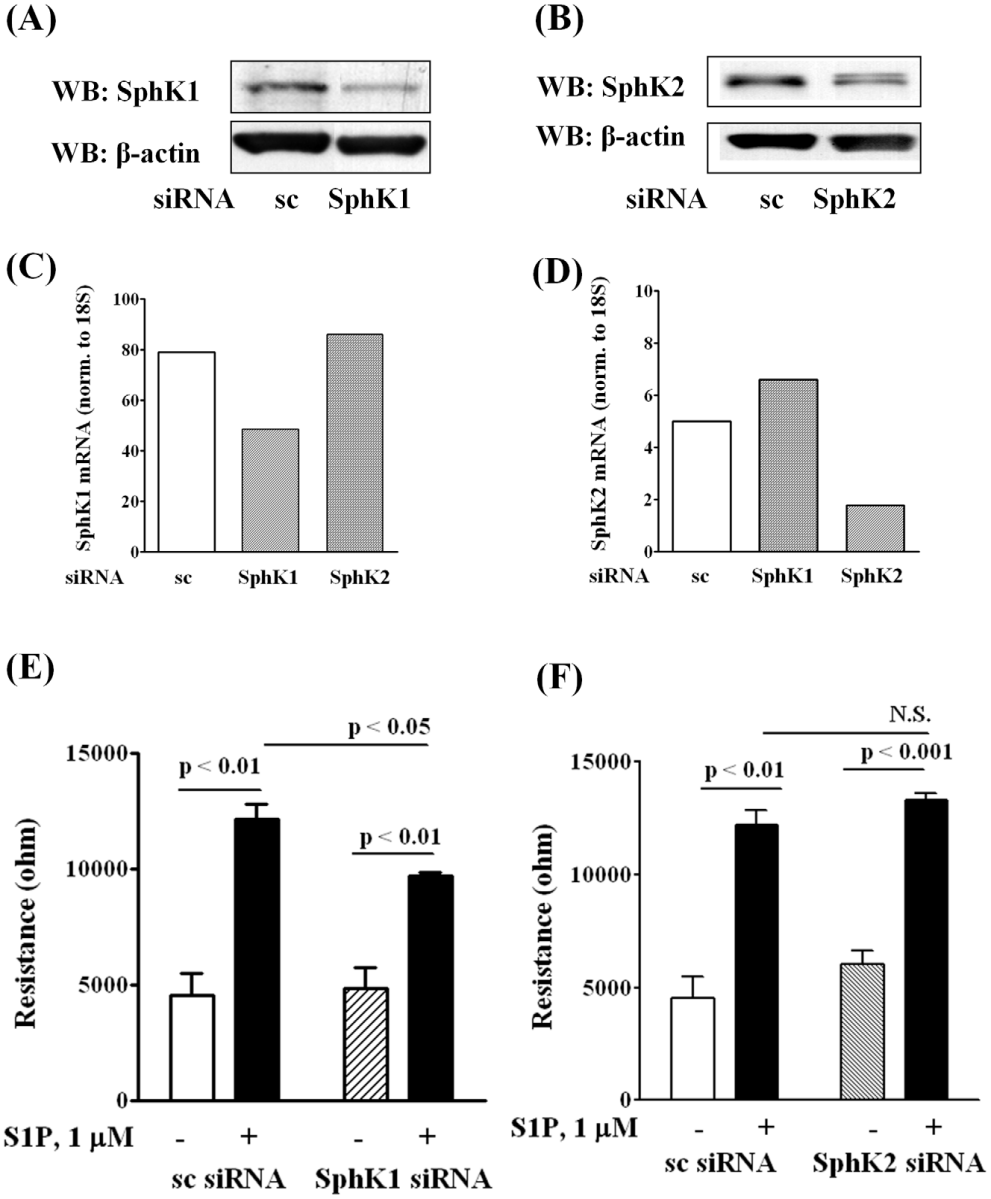
Silencing of SphK1, but not SphK2, decreases HPAEC migration by S1P in wound healing ECIS assay. HPAECs (∼50% confluence) grown on 35-mm dishes or on gold electrodes (10^–4^ cm^2^) were transfected with either scrambled siRNA or SphK1 siRNA or SphK2 siRNA (25 nM, 72 h), then starved for 3 h in 0.1% FBS in EBM-2 without growth factors. Cell lysates (20 µg total protein) were subjected to SDS-PAGE on 10% precast Tris-Glycine gels and Western blotted with anti-SphK1 (***A***) and SphK2 (***B***) antibody as described under “Experimental procedures”. Western blot is representative of three independent experiments. Total RNA was isolated from control and SphK1 siRNA (***C***) and SphK2 (***D***) transfected cells, and Real-time PCR was performed in a Light Cycler using SYBR Green QuantiTect. Control and transfected cells were wounded on the gold electrodes (***E*** and ***F***) as described under “Experimental Procedures” prior to S1P (1.0 µM) challenge. Measurement of transendothelial electrical resistance (TER) using an electrical cell substrate impedance-sensing system (ECIS) for 16 h after wounding the cells on the gold electrode and exposure to 1.0 µM S1P was carried out. Values are the mean ± S.E.M. for three independent experiments in triplicate.

Human pulmonary artery endothelial cells express SphK1 and SphK2 [19], and to determine the role of SphK1 and SphK2 in extracellular S1P (S1P_ext_)-induced migration, we employed an inhibitor of SphK to block S1P formation. Pretreatment of HPAECs with SphK inhibitor CII (10 µM) for 1 h, attenuated S1P (1 µM) mediated migration as determined by wound healing assay (**Fig. 1A**). The efficacy of CII was verified by measuring S1P formation in cells exposed to Sph. As shown in **Fig. 1B**, HPAECs that were pretreated with CII (10 µM) for 1 h showed diminished formation of S1P from Sph as compared to control cells. As CII blocks both SphK1 and SphK2 activities [39], role of SphK1 and SphK2 was investigated using siRNA specific for SphK1 or SphK2 to down-regulate the protein expression. Transfection of cells with SphK1 or SphK2 siRNA (50 nM) for 48 h knocked down >70% of SphK1 or SphK2 expression, as determined by Western blotting (**Fig. 2A and 2B**). The specificity of SphK1 or SphK2 siRNA is evidenced by down-regulation of mRNA expression for the specific SphK isoform without affecting the other (**Fig. 2C and 2D**). However, the SphK1 siRNA caused ∼40% reduction of SphK1 mRNA (**Fig. 2C**). Presumably, at least three reasons may account for this discordant mRNA and protein expression in human lung ECS. First, there are many complicated and varied post-transcriptional mechanisms involved in regulating mRNA into protein; second, proteins may differ in their *in vivo* half lives; and third, significant amount of error and noise in both protein and mRNA experiments. Down-regulation of SphK1 (50 nM), but not SphK2, for 48 h attenuated S1P-induced migration as determined by ECIS wound healing assay (**Fig. 2E and 2F**). Further, treatment of cells with SphK1 or SphK2 siRNA decreased intracellular S1P (S1P, fmol/nmol lipid P: scrambled siRNA, 348.0±13; SphK1 siRNA, 161±11; SphK2 siRNA, 196±11) as well as DHS1P levels (data not shown). Next, we tested the ability of exogenous sphingosine (Sph) to induce EC migration. Addition of exogenous Sph (1 µM) to wounded cells induced migration (16 h) that was significantly less compared to exogenous S1P (1 µM) (Closure of wounded area (%): Veh, 32±12; Sph, 56±9; S1P, 94±15). Similarly, exogenous Sph-induced cell migration as determined by ECIS wound healing assay was lower compared to S1P (data not shown). These results demonstrate that SphK1, but not SphK2, regulates S1P_ext_ mediated endothelial cell migration.

### Sphingosine-1-phosphate lyase siRNA or 4-Deoxypyridoxine Enhances Extracellular S1P-induced HPAEC Wound Healing and Migration

**Figure 3:**
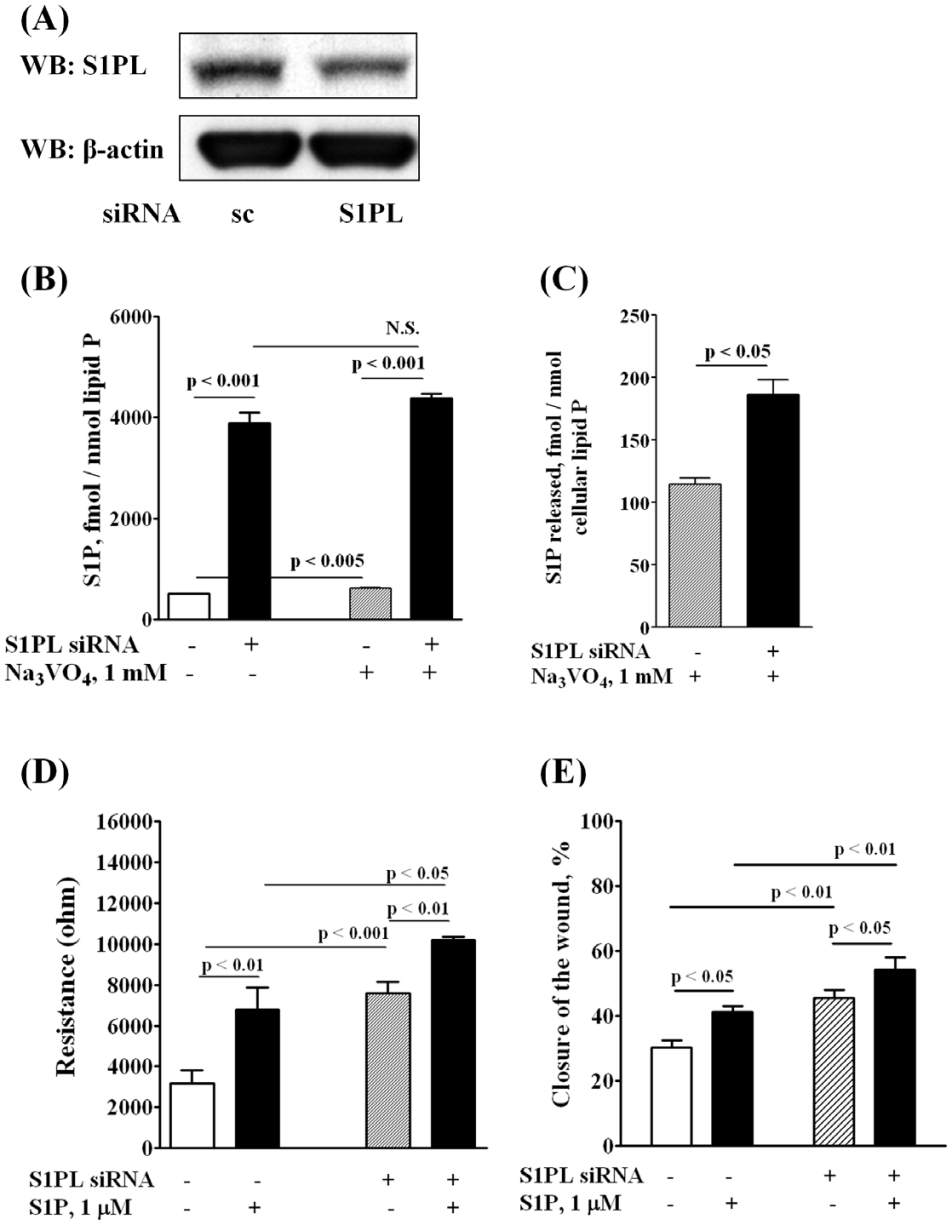
Silencing of S1PL increases intracellular S1P content in HPAECs and stimulates cell migration in an *in vitro* scratch and in a wound healing ECIS assay. HPAECs (∼50% confluence) grown on 35-mm dishes or on gold electrodes were transfected with either scrambled siRNA or S1PL siRNA (50 nM, 72 h), then starved for 3 h in 0.1% FBS in EBM-2 without growth factors. ***A*** - Cell lysates (20 µg total proteins) were subjected to SDS-PAGE and Western blotted with anti-S1PL antibody as described under “Experimental procedures”. Western blot is representative of three independent experiments. ***B,C*** – S1PL was silenced with siRNA (50 nM, 72 h) then intracellular *(* ***B*** *)* and extracellular *(* ***C*** *)* S1P content was determined by LC/MS/MS. Ortho-vanadate (1 mM) was applied 30 min before lipid extraction. S1P level in the medium was normalized per cellular phospholipid content. ***D*** – HPAECs (∼50% confluence) were transfected with either scrambled siRNA or S1PL siRNA (50 nM, 72 h) then wounded on the gold electrodes as described under “Experimental Procedures” prior to S1P (1.0 µM) challenge. Transendothelial electrical resistance (TER) was recorded using an electrical cell substrate impedance-sensing system (ECIS) for 16 h. Values are the mean ± S.E.M. for three independent experiments in triplicate. ***E -*** HPAECs (∼50% confluence) were transfected with either scrambled siRNA or S1PL siRNA (50 nM, 72 h) prior to scratching the cells for migration assay. Scratched cells were challenged with S1P (1.0 µM) for 16 h. The closure of the wound was evaluated as described under “Experimental Section” 16 h after the wounding of EC monolayer. The values are mean ± S.E.M. for three independent experiments in triplicates.

**Figure 4:**
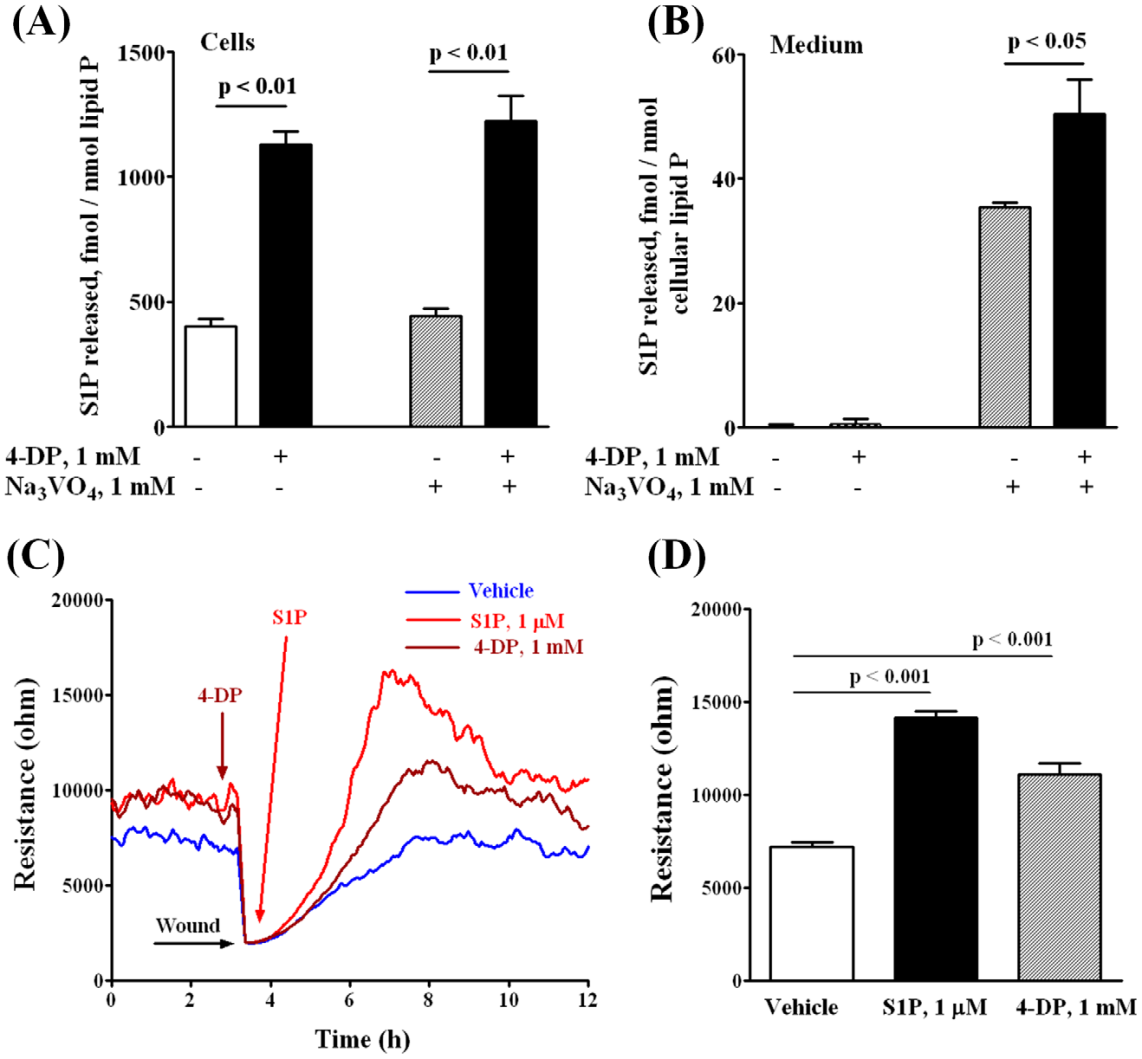
4-Deoxypyridoxine increases intracellular content of S1P in HPAECs and stimulates cell migration in a wound healing ECIS assay. HPAECs (∼90% confluence) grown on 35-mm dishes or on gold electrodes were starved for 3 h in 0.1% FBS in EBM-2 without growth factors and treated with 1 mM 4-DP for 6 h in the same medium. ***A, B*** – 4-DP increases intracellular content of S1P *(* ***A*** *)* and S1P release into the medium *(* ***B*** *)*. Ortho-vanadate was added 30 min before lipid extraction in a fresh medium. S1P content in cells and medium was determined by LC-MS/MS as described under “Experimental procedures”. *(* ***C, D*** *)* - Control and 4-DP-treated (1 mM for 30 min) cells were wounded on the gold electrodes as described under “Experimental Procedures” prior to S1P (1 µM) challenge. ***D*** shows the changes in TER (ohms) in vehicle and 4-DP or S1P-treated cells at 4 h after wounding. Values are the mean ± S.E.M. for three independent experiments.

Having established that blocking SphK1 decreases intracellular S1P levels and attenuates cell migration, next we investigated the role of sphingosine-1-phosphate lyase (S1PL), a pyridoxal phosphate dependent enzyme, in S1P_ext_ mediated cell motility and wound healing. S1PL expression was down-regulated with siRNA or activity was blocked with 4-deoxypyridoxine (4-DP), an analog of pyridoxine. Transfection of HPAECs with S1PL siRNA decreased S1PL mRNA (>80%) of control levels (data not shown) and protein expression (∼ 70%) (**Fig. 3A**). S1PL siRNA had no effect on mRNA or protein expression of SphK1 or SphK2 (data not shown). Further, knockdown of S1PL with siRNA increased accumulation of S1P (fmol/nmol of cellular lipid P: scrambled siRNA, 508±11; S1PL siRNA, 3887±211) (**Fig. 3B**) and dihydro S1P (DHS1P) (fmol/nmol of cellular lipid P: scrambled siRNA, 188±8; S1PL siRNA, 720±65) in cells and enhanced S1P-induced EC wound healing (**Fig. 3D**) and migration (**Fig. 3E**). Interestingly, treatment of cells with S1PL siRNA slightly stimulated basal migration of HPAECs in the absence of added exogenous S1P. In contrast to an increase of S1P or DHS1P in cells, we did not detect any appreciable accumulation of S1P or DHS1P in the medium. However, incubation of cells with sodium vanadate (1 mM for 1 h) increased S1P levels in the medium (**Fig. 3C**). However, under similar experimental conditions, DHS1P (fmol/nmol of cellular lipid P: scrambled siRNA, 20±8; S1PL siRNA, 18±5) levels in the medium were not altered. In parallel experiments, inhibition of S1PL activity with 4-DP (500 µM, 5 h) also increased S1P accumulation in cells (∼3 fold) as compared to control cells (fmol/nmol lipid P: control, 390±35; 4-DP, 1175±58) in the absence of sodium vanadate, and the addition of sodium vanadate had no effect on 4-DP-dependent S1P accumulation in cells (**Fig. 4A**). In the absence of sodium vanadate, no S1P release was detected in the medium with and without 4-DP; however, pretreatment of cells with sodium vanadate (1 mM, 1 h) enhanced S1P levels in the medium either in the presence or absence of 4-DP (**Fig. 4B**). 4-DP pretreatment also enhanced endothelial wound healing (**Fig. 4C and 4D**). In these experiments, wound healing by S1P_ext_ served as a positive control (**Fig. 4C and 4D**). These results show that increasing intracellular S1P levels by down-regulation of S1PL expression or blocking S1PL activity enhances S1P accumulation in cells and medium and stimulates basal wound healing in HPAECs.

### Role of Rac1 and IQGAP1 in [S1P]_ext_-induced HPAEC Migration

**Figure 5:**
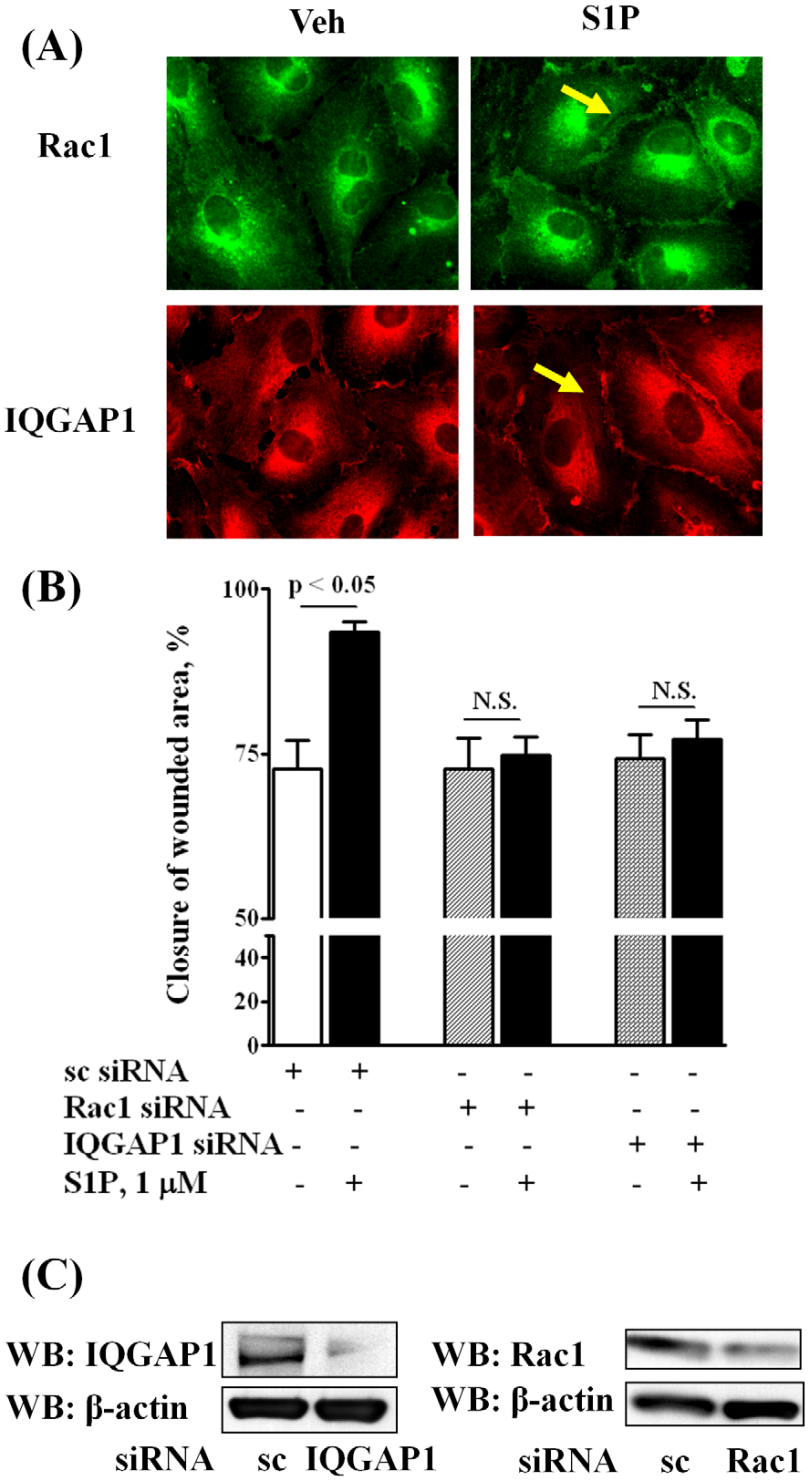
Silencing of Rac1 and IQGAP1 decreases HPAEC migration in an *in vitro* scratch assay. HPAECs (∼50% confluence) grown on 35-mm dishes or on cover slips were transfected with either scrambled siRNA or Rac1 siRNA or IQGAP1 siRNA (50 nM, 72 h), then starved for 3 h in 0.1% FBS in EBM-2 without growth factors. In ***A***, cells on cover slips were stimulated with 0.1% BSA-complexed S1P (1.0 µM) for 5 min, washed, fixed, permeabilized, probed with anti-Rac1 antibody and anti-IQGAP1 antibody, and examined by immunofluorescence microscopy using a ×60 oil objective. In ***B***, HPAECs (∼50% confluence) were transfected with either scrambled siRNA or Rac1 siRNA or IQGAP1 siRNA (50 nM, 72 h) prior to scratching the cells for migration assay. Scratched cells were challenged with S1P (1.0 µM) for 16 h. The values are means ± S.E.M. for three independent experiments each done in triplicates. ***C*** - Cell lysates (20 µg total proteins) were subjected to SDS-PAGE and Western blotted with anti-Rac1 and anti-IQGAP1 antibody as described under “Experimental procedures”. Western blot is representative of three independent experiments.

**Figure 6:**
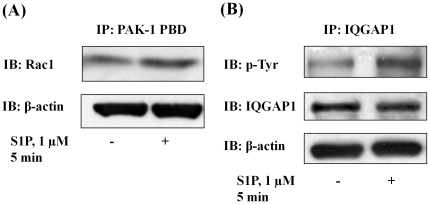
S1P induces Rac1 activation and IQGAP1 tyrosine phosphorylation in HPAECs. HPAECs (∼90% confluence) grown on 35-mm dishes were starved for 3 h in 0.1% FBS in EBM-2 without growth factors and treated with S1P (1 µM) for 5 min. Activated Rac1 was immunoprecipitated from total cell lysates (500 µg of total protein) from control and S1P (1 µM) treated cells using PAK-1 PBD agarose beads as described under “Experimental Procedures”. ***A***, Rac-1-GTP bound to PAK-1 PBD were separated by SDS-PAGE, transferred to nitrocellulose, and probed with anti-Rac1 antibody. ***B***, IQGAP1 was immunoprecipitated from total cell lysates (500 µg of total protein) from control and S1P (1 µM) treated cells using anti-IQGAP1 antibody. Immunoprecipitates were separated by SDS-PAGE, transferred to nitrocellulose, and probed with anti-p-Tyr antibody. Shown is a representative blot from three independent experiments.

**Figure 7:**
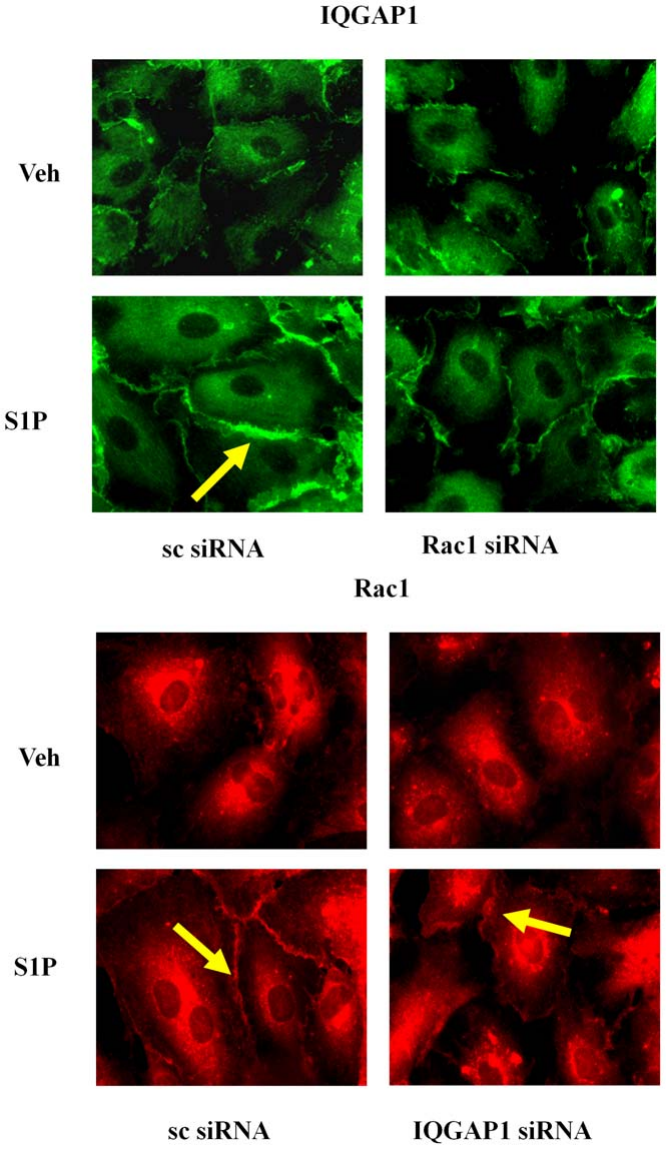
Rac1 siRNA attenuates translocation of IQGAP1 to the cell periphery. HPAECs (∼50% confluence) grown on coverslips were transfected with either scrambled siRNA or Rac1 siRNA or IQGAP1 siRNA (50 nM, 72 h), starved for 3 h in 0.1% FBS in EBM-2 without growth factors and treated with S1P (1 µM) for 5 min. Cells were washed, fixed, permeabilized, probed with anti-IQGAP1 or anti-Rac1 antibodies, and examined by immunofluorescence microscopy using a ×60 oil objective.

We have earlier demonstrated that S1P-induced activation of Rac1 regulates migration of HPAECs [12], and more recently identified a role for phospholipase D (PLD) dependent activation of IQGAP1 via Rac1 in hyperoxia-induced reactive oxygen species production in human lung endothelium [40]. To further characterize down-stream target(s) of Rac1 in cell migration, we investigated the role of IQGAP1, a IQ domain protein with a region containing sequence that has homology to RasGAP [41], in [S1P]_ext_ mediated wound healing. Exposure of HPAECs to S1P_ext_ (1 µM) for 15 min induced redistribution of Rac1 and IQGAP1 to cell periphery as evidenced by immunofluorescence microscopy (Relative Fluorescence Units: vehicle: Rac1, 100±23; IQGAP1, 100±29; S1P: Rac1, 250±38; IQGAP1 325±52) (**Fig. 5A**). As shown in **Fig. 5B**, down-regulation of Rac1 or IQGAP1 with siRNA (50 nM, 48 h), compared to scrambled siRNA (50 nM, 48 h), attenuated [S1P]_ext_-induced cell motility. The efficacy of Rac1 and IQGAP1 siRNA was evaluated by Western blotting of Rac1 and IQGAP1 that showed greater than 70% knockdown of the protein expression (**Fig. 5C**). Activation of Rac1 and IQGAP1 by S1P_ext_ was verified by precipitation of Rac1 bound to GTP with PAK-1 PBD beads and IQGAP1 tyrosine phosphorylation, respectively (**Fig. 6A and 6B**). Next, we investigated whether Rac1 was upstream of [S1P]_ext_-induced IQGAP1 activation. HPAECs were transfected with Rac1 or IQGAP1 siRNA (50 nM, 48 h) and exposed to [S1P]_ext_ (1 µM) for 30 min. As shown in **Fig. 7**, Rac1 siRNA almost completely blocked S1P-induced IQGAP1 redistribution to cell periphery while IQGAP1 siRNA had no effect on [S1P]_ext_-dependent Rac1 redistribution to cell periphery. These data suggest a potential involvement of Rac1 and IQGAP1 in [S1P]_ext_-induced HPAEC migration, and IQGAP1 as a down-stream target of Rac1.

### SphK1 siRNA, but not SphK2 siRNA, Attenuates [S1P]_ext_-induced Rac1 and IQGAP1 Redistribution to Cell Periphery

**Figure 8:**
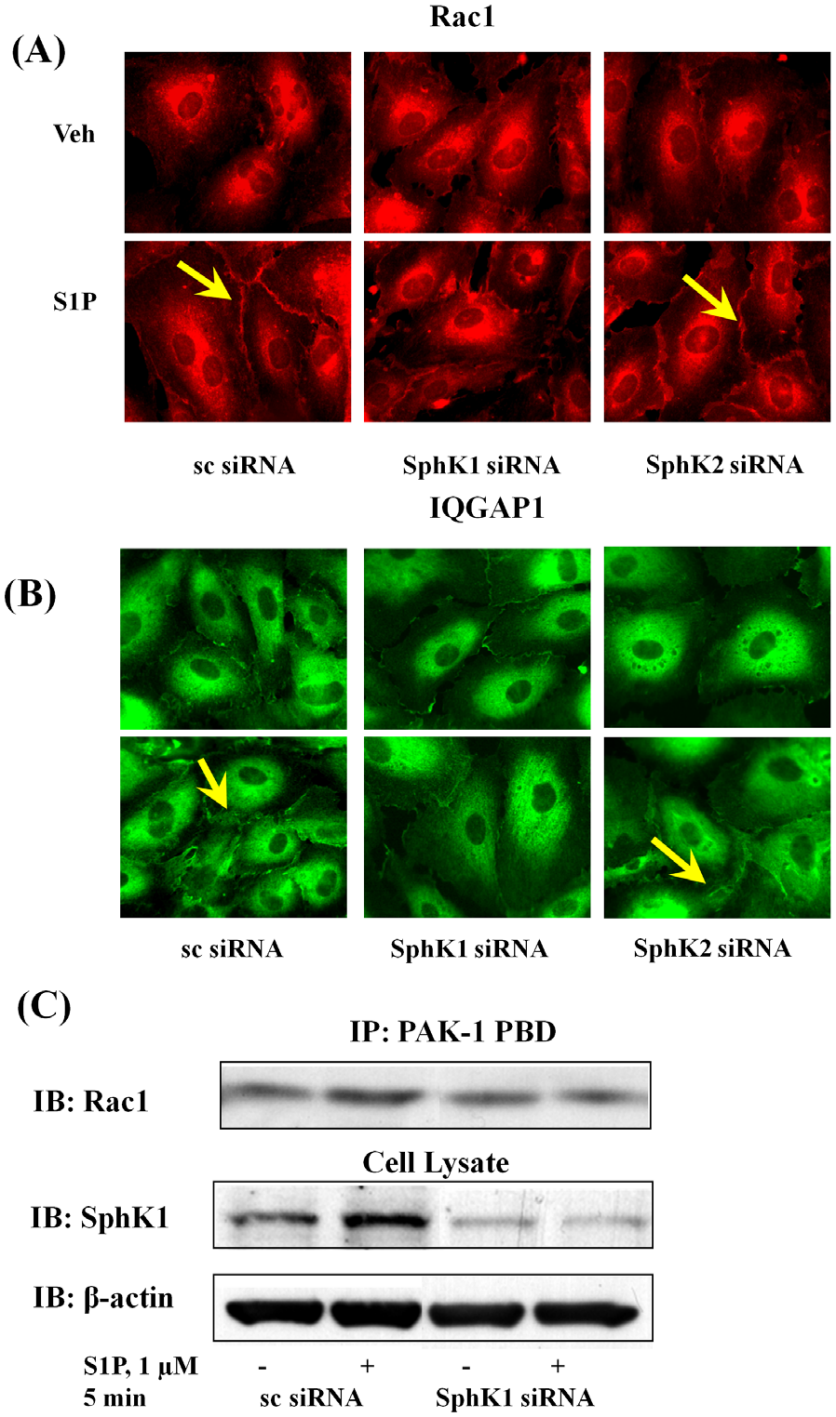
SphK1 siRNA, but not SphK2 siRNA, attenuates Rac1 activation and IQGAP1 translocation to cell periphery of HPAECs. HPAECs (∼50% confluence) grown on 35-mm dishes or cover slips were transfected with either scrambled siRNA or SphK1 siRNA or SphK2 siRNA (25 nM, 72 h), then starved for 3 h in 0.1% FBS in EBM-2 without growth factors. Cells on cover slips were stimulated with 0.1% BSA-complexed S1P (1.0 µM) for 5 min, washed, fixed, permeabilized, probed with anti-Rac1 antibody (***A***) and anti-IQGAP1 (***B***) antibody, and examined by immunofluorescence microscopy using a ×60 oil objective. In ***C***, activated Rac1 was immunoprecipitated from total cell lysates (500 µg of total protein) from control, transfected and S1P (1.0 µM) treated cells using PAK-1 PBD agarose beads as described under “Experimental Procedures”. Rac-1-GTP bound to PAK-1 PBD were separated by SDS-PAGE, transferred to nitrocellulose, and probed with anti-Rac1 antibody. Shown is a representative blot from three independent experiments.

Having established a role for Rac1 and IQGAP1 in [S1P]_ext_-induced cell migration, we next investigated the effect of down-regulation of SphK1 or SphK2 with siRNA on basal and [S1P]_ext_-induced redistribution of Rac1 and IQGAP1. HPAECs were transfected with scrambled, SphK1 or SphK2 siRNA (50 nM, 48 h) prior to S1P challenge, and as shown in **Fig. 8A and 8B**, SphK1 siRNA, but not scrambled or SphK2 siRNA, attenuated [S1P]_ext_-induced Rac1 and IQGAP1 translocation to cell periphery. Further, down-regulation of SphK1 with siRNA attenuated S1P-induced Rac1 activation as determined by precipitation of Rac1 bound to GTP with PAK-1 PBD beads (**Fig. 8C**). These results suggest a role for SphK1 in S1P_ext_-induced translocation of Rac1 and IQGAP1 to cell periphery in HPAECs.

### Down-regulation of S1PL with siRNA or Inhibition of S1PL Activity with 4-Deoxypyridoxine Stimulates Rac1 and IQGAP1 Redistribution to Cell Periphery

**Figure 9:**
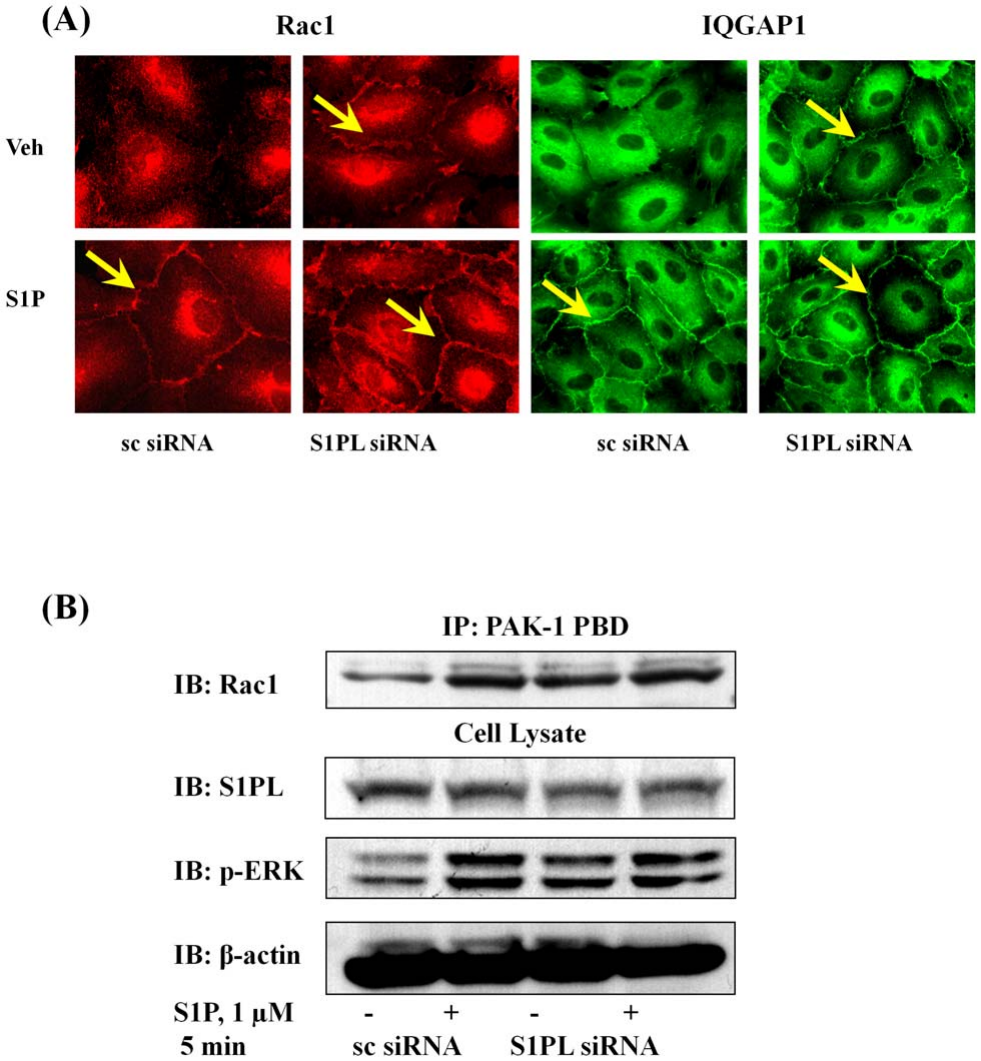
Silencing of S1PL stimulates Rac1 activation and IQGAP1 translocation to cell periphery of HPAECs. HPAECs (∼50% confluence) grown on 35-mm dishes or cover slips were transfected with either scrambled siRNA or S1PL siRNA (50 nM, 72 h), then starved for 3 h in 0.1% FBS in EBM-2 without growth factors. ***A -*** Cells on cover slips were stimulated with 0.1% BSA-complexed S1P (1.0 µM) for 5 min, washed, fixed, permeabilized, probed with anti-Rac1 antibody and anti-IQGAP1 antibody, and examined by immunofluorescence microscopy using a ×60 oil objective. ***B*** - Activated Rac1 was immunoprecipitated from total cell lysates (500 µg of total protein) from control, transfected, and S1P (1.0 µM) treated cells using PAK-1 PBD agarose beads as described under “Experimental Procedures”. Rac-1-GTP bound to PAK-1 PBD was separated by SDS-PAGE, transferred to nitrocellulose, and probed with anti-Rac1 antibody. Shown is a representative blot from three independent experiments.

**Figure 10:**
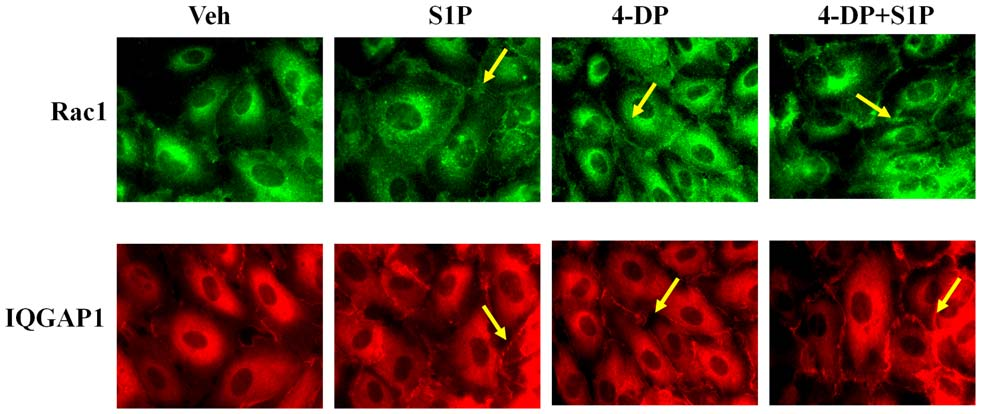
4-Deoxypyridoxine stimulates translocation of Rac1 and IQGAP1 to the cell periphery. HPAECs (∼90% confluence) grown on coverslips were starved for 3 h in 0.1% FBS in EBM-2 without growth factors then treated with 4-DP (1 mM) for 6 h, with or without final treatment with S1P (1 µM) for 5 min. Cells were washed, fixed, permeabilized, probed with anti-Rac1 antibody and anti-IQGAP1 antibody, and examined by immunofluorescence microscopy using a ×60 oil objective. Shown is a representative micrograph from three independent experiments.

We next investigated the effect of knockdown of S1PL expression or inhibition of S1PL activity on basal and [S1P]_ext_-induced redistribution of Rac1 and IQGAP1 to cell periphery and activation of Rac1. HPAECs either on glass coverslips or 100 mm dishes were transfected with scrambled or S1PL siRNA (50 nM, 48 h) prior to exposure to media alone or media containing S1P (1 µM). As shown in **Fig. 9A**, knockdown of S1PL with siRNA enhanced redistribution of Rac1 and IQGAP1 to cell periphery under basal condition (without exogenous S1P). Further, silencing of S1PL with siRNA dramatically increased Rac1 activation under basal condition that was similar to [S1P]_ext_ mediated Rac1 activation (**Fig. 9B**). In S1PL down-regulated cells with siRNA, stimulation with [S1P]_ext_ did not further enhance the basal Rac1 activation (**Fig. 9B**). In parallel experiments, Inhibition of S1PL with 4-DP (500 µM) resulted in a similar activation of Rac1 and IQGAP1 in the absence of [S1P]_ext_ addition to HPAECs (**Fig. 10**). Taken together, these results demonstrate that increased S1P levels in cells of S1PL siRNA or 4-DP treated cells stimulate Rac1 and IQGAP1 that is independent of [S1P]_ext_.

### Pertussis Toxin Attenuates [S1P]_ext_ and S1PL siRNA Mediated Redistribution of Rac1 and IQGAP1 in HPAECs

**Figure 11:**
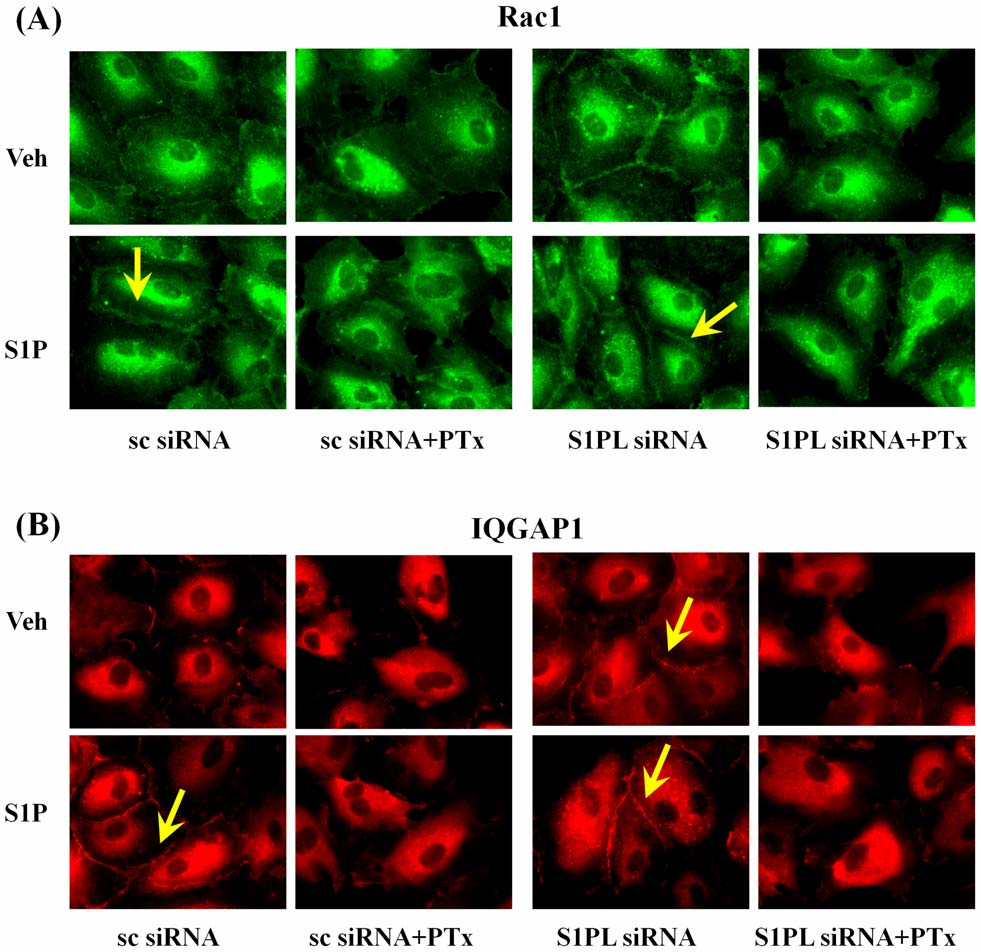
Pertussis toxin attenuates translocation of Rac1 and IQGAP1 to cell periphery induced by S1P or by silencing of S1PL. HPAECs (∼50% confluence) grown on cover slips were transfected with either scrambled siRNA or S1PL siRNA (50 nM, 72 h), then treated with Pertussis toxin (100 ng/ml) for 16 h in 2% FBS in EBM-2 without growth factors. Cells were stimulated with 0.1% BSA-complexed S1P (1.0 µM) for 5 min, washed, fixed, permeabilized, probed with anti-Rac1 (***A***) or anti-IQGAP1 (***B***) antibodies, and examined by immunofluorescence microscopy using a ×60 oil objective.

Our earlier studies demonstrated that S1P_1_ is the predominant receptor expressed in HPAECs and the involvement of Gi in human lung EC migration by transducing signals initiated by S1P activation of S1P_1_ [12]. Here, we investigated the effect of pertussis toxin (PTx) on [S1P]_ext_ - and S1PL siRNA-induced cell migration. HPAECs transfected with scrambled or S1PL siRNA (50 nM, 48h) were treated with PTx (100 ng/ml, 16 h) prior to challenge with media alone or media plus [S1P]_ext_ (1 µM). PTx significantly blocked basal-, [S1P]_ext_- and S1PL siRNA-mediated redistribution of Rac1 (**Fig. 11A**) and IQGAP1 (**Fig. 11B**). These results show that the activation of Rac1 and IQGAP1 due to S1P accumulation by S1PL siRNA is sensitive to PTx suggesting involvement of Gi and S1P receptors.

### Antagonist of S1P_1 and 3_, but not S1P_2_, Block S1PL siRNA- or 4-Deoxypyridoxine- Mediated basal Cell Migration

**Figure 12:**
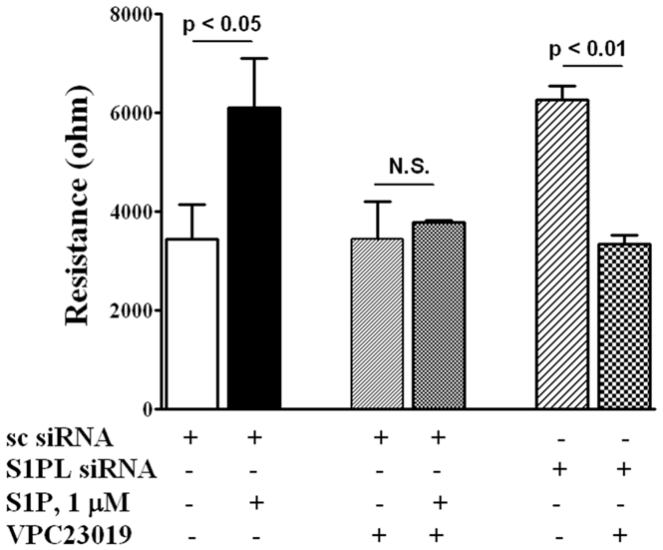
Antagonist of S1P_1_ and S1P_3_, but not S1P_2_, blocks HPAEC migration induced by S1P and S1PL silencing in a wound healing ECIS assay. HPAECs (∼50% confluence) grown on gold electrodes were transfected with either scrambled siRNA or S1PL siRNA (50 nM, 72 h), then starved for 3 h in 0.1% FBS in EBM-2 without growth factors. Control and transfected cells were wounded on the gold electrodes as described under “Experimental Procedures” prior to VPC23019 (10 µM for 15 min) and following S1P (1.0 µM) challenge. Transendothelial electrical resistance (TER) was recorded for 16 h. Values are the mean ± S.E.M. for three independent experiments each performed in triplicates.

Having established a role for SphK1 and S1PL in S1P-mediated migration of HPAECs, we next studied the role of *S1P_1-3_* on basal, [S1P]_ext_-, S1PL siRNA - or 4-DP-induced cell migration. Stimulation of EC migration by [S1P]_ext_ or by silencing the S1PL was blocked by VPC23019 (10 µM), an antagonist of *S1P_1_*_ and *3*_ [42] (**Fig. 12**). In contrast to VPC23019, pretreatment of cells with JTE-013, an antagonist of *S1P_2_*, had no significant effect on migration of HPAECs initiated by [S1P]_ext_ or by S1PL inhibition (data not shown). These results suggest that stimulation of cell migration by [S1P]_ext_ or intracellularly generated S1P by S1PL siRNA requires coupling to *S1P_1 and/or 3_* in HPAECs.

### S1PL siRNA or 4-Deoxypyridoxine-Mediated Cell Migration and Redistribution of Rac1 and IQGAP1 Involve Secretion of Intracellular S1P

**Figure 13:**
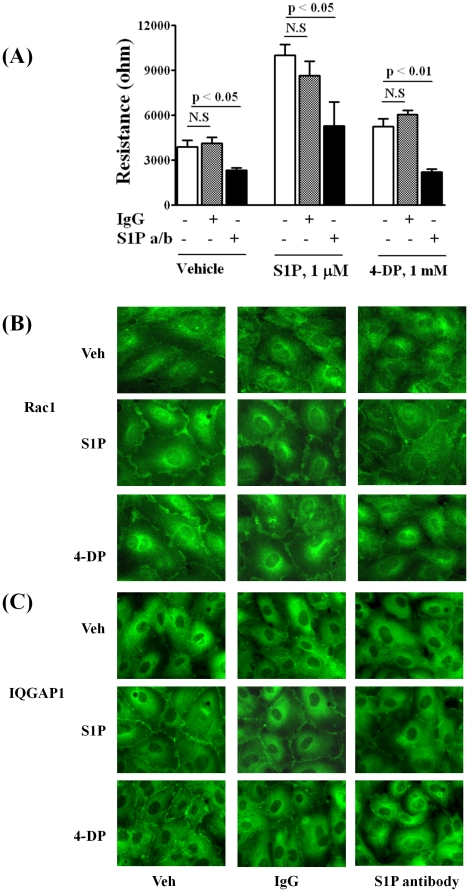
S1P antibodies decrease S1P- and 4-Deoxypyridoxine-induced HPAEC migration. HPAECs (∼90% confluence) grown on gold electrodes or coverslips were starved for 3 h in 0.1% FBS in EBM-2 without growth factors. ***A*** -Cells were treated with 4-DP (1 mM for 30 min) then wounded on the gold electrodes as described under “Experimental Procedures” prior to the addition of isotype-matched control IgGk1 or anti-S1P antibody (150 µg/ml) and S1P (1 µM) challenge. Transendothelial electrical resistance was recorded for 16 h. Values are the mean ± S.E.M. for three independent experiments each performed in triplicate. ***B,C*** - Cells on cover slips were treated with 4-DP (1 mM) and anti-S1P antibody for 6 h, then stimulated with 0.1% BSA-complexed S1P (1.0 µM for 5 min), washed, fixed, permeabilized, probed with anti-Rac1 antibody (***B***) or anti-IQGAP1 antibody (***C***), and examined by immunofluorescence microscopy using a ×60 oil objective. Shown is a representative micrograph from several independent experiments.

Having established that S1PL siRNA or 4-DP treatment increases S1P levels in cells and stimulates endothelial cell motility, we next evaluated whether these effects were mediated by an autocrine pathway of S1P secretion and signaling via S1P_1 and/or 3_ by using a highly specific anti-S1P monoclonal antibody (mAb) [33], [34]. This S1P specific antibody, at a concentration of 150 µg/ml, has been shown to effectively neutralize about 2 µM of extracellular S1P released or added into the condition medium [33]. As shown in **Fig. 13A**, addition of the anti-S1P mAb (150 µg/ml) to the medium abrogated S1P_ext_ or 4-DP induced cell migration measured by ECIS wound healing assay. Addition of an isotype-matched mouse IgGk1 (150 µg/ml) to the medium, under same experimental condition, had no significant effect on [S1P]_ext_ - or 4-DP-induced wound healing of HPAECs. (**Fig. 13A**). Similarly, anti-S1P mAb effectively blocked [S1P]_ext_- or 4-DP-dependent translocation of Rac1 (**Fig. 13B**) and IQGAP1 (**Fig. 13C**) to cell periphery. These data show that cellular responses due to elevation of cellular S1P by S1PL siRNA or 4-DP, in part, are mediated by the “inside-out” signaling mechanism and ligation to S1P receptors on the cell surface.

## Discussion

Endothelial cell migration is a key component of angiogenesis and an important physiological response for normal vascular development; however, is abnormal in pathophysiological conditions such as age-related wet macular degeneration and tumor angiogenesis. Among the plethora of growth factors that regulate endothelial cell angiogenic responses, S1P is probably the most potent [9] and is the ligand for the family of S1P_1–5_ G-protein coupled receptors [2]–[7]. While evidence for extracellular action of S1P via its receptors in cell migration is compelling, it is unclear if intracellular generation of S1P is necessary for [S1P]_ext_ function. In this study, we have evaluated the role of intracellular S1P generation and the balance between its synthesis and degradation in regulating [S1P]_ext_-mediated endothelial cell migration. The results presented here provide evidence that modulation of SphK1 and S1PL, enzymes involved in the conversion of Sph to S1P and breakdown of S1P to hexadecenal and ethanolamine phosphate, respectively, exhibit a profound effect on cell migration. Our results further suggest that exogenous S1P is not sufficient by itself to exert a full effect on EC migration as evidenced by data showing that inhibiting SphK1 with either CII or down-regulation of SphK1 with siRNA attenuated S1P-induced migration.

Earlier studies have shown that S1P-induced EC migration is regulated by several distinct signaling pathways and depends on the cell type, type of S1P receptor, and concentrations of S1P. S1P stimulates cell migration through extracellular ligation of its receptors, S1P_1_ and S1P_3_ coupled to G_αi_, G_q_ or G_12/13_ and activation of Rac, MAPKs and PI3K [43]. In contrast to S1P_1_ and S1P_3,_ S1P_2_ seems to have an opposing effect on EC migration as blocking S1P_2_ up-regulated S1P-induced migration in vascular ECs and smooth muscle cells [44], [45]. Interestingly, S1P_1_ is essential for embryogenesis and vessel maturation as S1P_1_ −/− null mice die at day 14 of embryogenesis [46]. We have earlier demonstrated in human lung endothelial cells that S1P signals through S1P_1_ and G_i_ to activate PKC-ε and subsequently, a PLD2→PKC-ζ→Rac1 cascade to stimulate migration [12]. Although, we have demonstrated a role for PI3K in EC motility [47], blocking PI3K with LY294002 had no effect on S1P-induced PLD activation [12] indicating PLD- and PI3K-dependent pathways of S1P mediated cell motility.

An important finding of the present study is that endogenous S1P production is essential for [S1P]_Ext_ mediated EC migration. In ECs and other cell types, SphK1 and/or SphK2 catalyze the phosphorylation of sphingosine to S1P and accumulation of S1P is a balance between synthesis and degradation mediated by S1PL, lipid phosphate phosphatases and S1P phosphatases [19]–[22]. We observed that blocking SphK1 reduced intracellular S1P generation and S1P-induced cell migration and resistance while knockdown or attenuation of S1PL enhanced S1P levels and potentiated migration (**Figs. 1** **,** **2** **,** **3**). These results are in agreement with our earlier study demonstrating the ability of human lung ECs to convert exogenously added S1P to sphingosine by LPPs on the cell surface, followed by the rapid uptake of sphingosine into the cell and conversion to S1P by SphK1 [19]. Thus, in addition to its extracellular action, endogenous S1P generation is essential for sustained S1P-mediated cell migration likely due to a recycling effect whereby S1P is initially dephosphorylated and then sent back to SphK1 for phosphorylation to S1P and eventual release. Although human lung ECs rapidly take up exogenous sphingosine and converts it to S1P in the cell, EC migration is more pronounced with exogenous S1P (1 µM) as compared to exogenous sphingosine (1 µM). This discordant effect of exogenous S1P and sphingosine was also observed in changes in electrical resistance and phosphorylation of ERK1/2 in lung ECs described earlier [31].

Interestingly, S1P delivered as a liposome, induced non-directional cell movement, in contrast to directional cell motility by extracellular S1P in L6, COS7 and SH-SY5Y cell types [48] indicating translocation of SphK1 to certain specialized areas of the cell such as membrane ruffles in the formation of cell polarity. However, the mechanism(s) of extracellular vs. liposomal S1P action on formation of cell polarity and random migration as well as role for S1P receptors were not addressed. In a recent study, a role for SphK1 in nitric oxide-mediated human endothelial cell migration and tube formation was demonstrated; however, the contribution of endogenous S1P was not demonstrated [49]. Similarly, TGF-β stimulated collagen production in cardiac fibroblasts is critically dependent on intracellular S1P generated by SphK1 [50]. While many studies including the results presented here strongly support a role for SphK1 in cellular functions such motility and collagen production, a recent report suggests that SphK2 deficiency contributes to reduced S1P accumulation in lymphoid tissues and attenuated T and B cell lymphopenia [51]. Thus, role of SphK1 and/or SphK2 may vary in different cell types and tissues.

Because our results show a requirement for intracellularly generated S1P in extracellular S1P-mediated cell migration, it was important to delineate the mechanism. S1P generated inside the cell can act as an intracellular second messenger as evidenced by increased DNA synthesis [52] and calcium mobilization from internal stores [2], [3] or signal ‘inside-out’ via S1P receptors [25]. However, the intracellular target(s) mediating these S1P responses have not been well characterized. Recently, interaction between SphK2/S1P and histone deacetylases in breast cancer cells [15] and S1P as a missing co-factor for E3 ubiquitin ligase TRAF2 in HEK 293 cells [28] were shown. Our results, using a two prong approach of pretreatment with PTx or anti-S1P antibody, show that intracellular S1P regulates cell motility through an ‘inside-out’ signaling pathway. The increased redistribution of Rac1 and IQGAP1 due to exogenous S1P addition or down-regulation of S1PL was PTx sensitive suggesting release and signaling of S1P from outside the cell involving S1P receptors. Blocking of S1P_1_ and S1P_3_ activation by VPC23019 attenuated S1P as well as S1PL siRNA mediated increase of endothelial electrical resistance confirming involvement of these two S1P receptors in barrier regulation. Further, the ability of the anti-S1P antibody to neutralize S1P's effects on endothelial layer resistance (**Fig. 13A**) confirms an extracellular signaling role for S1P and is consistent with the ability of the anti-S1P antibody to block the S1P/TGF-β axis with regard to collagen expression in cardiac fibroblast [50]. Interestingly, treatment of cells with anti-S1P antibody slightly reduced basal electrical resistance even in the absence of exogenous S1P or 4-DP (**Fig. 13A**). The ‘inside-out’ signaling was further substantiated in studies where over-expression of LPP-1 wild type attenuated S1PL siRNA- or 4-DP-mediated increase in electrical resistance (data not shown). This is the first time that S1P has been shown to utilize an ‘inside-out’ signaling mechanism in cell motility. These findings suggest a role for basal S1P release through ‘inside-out’ signaling in the maintenance of endothelial barrier integrity. Release of intracellularly generated S1P to cell media may involve members of the ABC transporter family, ABCC1 [29], ABCA1 [53] and ABCG2 [54]; however, other mechanisms must be operating as genetic knockouts of ABCC1, ABCA1, and ABCA7 do not result in lowered plasma S1P levels. Recently, Spns2 protein has been identified as an S1P transporter in zebra fish [55]. At present it is unclear if ABC transporter protein(s) or Spns2 or both are involved in regulating ‘inside-out’ S1P secretion in human lung endothelial cells that needs to be determined. 4-DP treated cells mitigated Rac1 and IQGAP1 redistribution to cell periphery and changes in electrical resistance (**Figure 13**). This demonstrates that anti-S1P antibody is able to neutralize exogenous or endogenous S1P secreted to cell culture media and prevent S1P mediated ‘inside-out’ signaling. The concentration of anti-S1P antibody (150 µg/mL) that effectively blocked extracellular or secreted S1P-induced Rac1/IQGAP1 redistribution to cell periphery and increase in electrical resistance measurements suggests a substantial release of S1P (µM) into the extracellular compartment. Our work also demonstrates that the released S1P is likely rapidly degraded after triggering S1P receptor-mediated signaling events by the action of phosphatases that produce Sph for recycling into a new pool of releasable S1P by providing substrate for intracellular SphKs 1 and 2.

## Materials and Methods

### Standards and Reagents

S1P, DHS1P, a 17-carbon analog of S1P (C17-S1P) were obtained from Avanti Polar Lipids (Alabaster, AL, USA). Rac1 activation assay kit was obtained from Upstate (Temecula, CA, USA). Formaldehyde (36.5%) was obtained from Sigma (St. Louis, MO, USA). Lysis buffer was purchased from Cell Signaling Technology Inc. (Danvers, MA, USA). Protease inhibitor cocktail tablets (EDTA-free Complete) were from Roche Diagnostics (Indianapolis, IN, USA). Aprotinin and phosphatase inhibitor cocktail 1 were from Sigma-Aldrich (St. Louis, MO, USA).

*Inhibitors—*VPC23019 were obtained from Avanti Polar Lipids (Alabaster, AL, USA). Pertussis toxin was purchased from Calbiochem (La Jolla, CA, USA). 4-Deoxypyridoxine was purchased from Sigma (St. Louis, MO, USA). SphK inhibitor, 2-(p-hydroxyanilino)-4-(p-chlorophenyl)thiazole (CII), was obtained from Cayman (Ann Arbor, MI, USA).

### siRNA

Scrambled and target siRNA for SphK1, SphK2, and S1P_1_ were obtained from Dharmacon (Lafayette, CO, USA). siRNA for S1PL, Rac1 and IQGAP1 were from Santa-Cruz Biotechnology, Inc. (Santa Cruz, CA, USA).

### Antibodies

Anti-IQGAP1, anti-phospho-ERK, and anti-S1P_1_ antibody were purchased from Santa Cruz Biotechnology, Inc. (Santa Cruz, CA, USA). Anti-S1P_2_, anti-S1P_3_, anti-S1P_4_, anti-S1P_5_ antibodies were purchased from Exalpha Biological Inc. (Maynard, MA, USA); anti-SphK1 was obtained from Abcam, anti-SphK2 antibody were kindly provided by Dr. Taro Okada from Department of Molecular and Cellular Biology (Kobe University Graduate School of Medicine, Japan). Anti-Rac1 antibody was from BD Biosciences Pharmingen (San Jose, CA, USA); anti-β-actin antibody was from Sigma (St. Louis, MO, USA). Murine anti-S1P monoclonal antibodies (LT1002) and control isotype-matched IgGk1 antibodies came from Lpath, Inc. [33], [34].

### Cell Culture

HPAECs (passage number 3) were purchased from Cambrex Inc. (Walkersville, MD, USA) and cultured in complete endothelial growth medium (EGM)-2 medium [19]. The cells (passage number 5-8) in 35-mm or 100-mm dishes or glass coverslips were used for all the experiments.

### Endothelial Cell Migration

HPAEC were cultured in 12- or 6-well plates to ∼95% confluence and then starved in the serum-free EGM-2 medium for 1–3 h or in EBM-2 medium containing 1% FBS for 18–24 h. The cell monolayer was wounded by scratching across the monolayer with a 10 µl standard sterile pipette tip. The scratched monolayer was rinsed twice with serum-free medium to remove cell debris and incubated with varying concentrations of S1P. The area (∼1 cm^2^ total) in a scratched area was recorded at 0 h and 16–24 h using a Hamamatsu digital camera connected to the Nikon Eclipse TE2000-S microscope with ×10 objective and MetaVue software (Universal Imaging Corp., PA, USA) images were analyzed by the Image J software. The effect of S1P and other agents on endothelial cell migration/wound healing was quantified by calculating the percentage of the free area not occupied by cells compared to an area of the initial wound that was defined as closure of wounded area.

### Electrical Cell Substrate Impedance Sensing (ECIS) Assay

HPAEC were cultured in 8-well ECIS electrode arrays (8W1E, Applied Biophysics, NY, USA) to ∼95% confluence and starved in the serum-free EBM-2 medium for 1–3 h. An elevated field (3 V at 40,000 Hz for 10 sec) was applied to wound the cells on the electrode. S1P (1 µM) was added and endothelial wound healing was monitored for 10–20 h by measuring the transendothelial electrical resistance using the ECIS equipment [35], [36]. In all experiments S1P was complexed with 0.1% BSA.

### Rac1 Activation Assay

HPAECs were cultured in 100 mm dishes to ∼50% confluence for siRNA transfection or to ∼95% confluence for adenoviral infection or inhibitor treatment. Cells were starved in EBM-2 medium containing 0.1% FBS for 3 h prior to stimulation with S1P for 5–15 min, cell lysates were subjected to immunoprecipitation with PAK-1 PBD and Rac1 activation was evaluated using the Rac1 Activation Assay Kit as per the manufacturer's instruction (Upstate, Temecula, CA, USA).

### Western Blot Analysis

HPAECs were cultured in 6-well plates or 60 mm dishes to ∼95% confluence and starved for 3 h in EBM-2 medium containing 0.1% FBS. Cells were stimulated with S1P (100–1000 nM) for 5–60 min, washed with PBS and lysed with 100–300 µl lysis buffer containing 20 mM Tris-HCl (pH 7.5), 150 mM NaCl, 1 mM Na_2_EDTA, 1 mM EGTA, 1% Triton X-100, 2.5 mM sodium pyrophosphate, 1 mM β-glycerophosphate, 1 mM Na_3_VO_4_, 1 µg/ml leupeptin, 1 µg/ml aprotinin and protease inhibitors from EDTA-free Complete tablets (Roche Applied Science, Indianapolis, IN, USA). Cell lysates were cleared by centrifugation at 10,000×g for 10 min, and boiled with the Laemmli sample buffer for 5 min. Cell lysates (20–30 µg protein) were separated on 10% or 4–20% SDS-PAGE, transferred to PVDF membranes, blocked in TBST containing 5% BSA prior to incubation with primary antibody (1∶1000 dilution) overnight. After blocking, washing and incubation with appropriate secondary antibody, blots were developed using an ECL chemiluminescence kit. Western blots were scanned by densitometry and integrated density of pixels in identified areas was quantified using Image Quant version 5.2 software (Molecular Dynamics, CA, USA).

### Immunofluorescence Microscopy

HPAECs grown on coverslips (18-mm) or chamber slides were starved for 3 h in EBM-2 containing 1% FBS prior to treatment with S1P (1 µM) for 5–15 min. Cells were fixed in 3.7% formaldehyde in PBS for 10 min, washed three times with PBS, permeabilized with methanol for 4 min at −20° C, blocked with 2% BSA in TBST, incubated for 1 h with appropriate primary antibody (1∶200 dilution), washed with TBST, and stained for 1 h with secondary antibody (1∶200 dilution) in TBST containing 2% BSA. Cells were examined using a Nikon Eclipse TE2000-S immunofluorescence microscope and a Hamamatsu digital camera with ×60 oil immersion objective and Meta Vue software.

### RNA Isolation and Real Time RT-PCR

Total RNA was isolated from HPAECs grown on 35-mm dishes using TRIzol® reagent according to the manufacturer's instruction. iQ SYBR Green Supermix was used to do the real time measurements using iCycler by BioRad. 18S (sense, 5′-GTAACCCGTTGAACCCCATT-3′, and antisense, 5′- CCATCCAATCGGTAGTAGCG-3′) was used as a housekeeping gene to normalize expression. The reaction mixture consisted of 0.3 µg of total RNA (target gene) or 0.03 µg of total RNA (18S rRNA), 12.5 µl of iQ SYBR Green, 2 µl of cDNA, 1.5 µM target primers, or 1 µM 18S rRNA primers, in a total volume of 25 µl. For all samples, reverse transcription was carried out at 25°C for 5 min, followed by cycling to 42°C for 30 min and 85°C for 5 min with iScript cDNA synthesis kit. Amplicon expression in each sample was normalized to its 18S rRNA content. The relative abundance of target mRNA in each sample was calculated as 2 raised to the negative of its threshold cycle value times 10^6^ after being normalized to the abundance of its corresponding 18S rRNA (housekeeping gene), (2^-(primer Threshold Cycle)^/2^-(18 S Threshold Cycle)^x 10^6^). All primers were designed by inspection of the genes of interest using Primer 3 software. Negative controls, consisting of reaction mixtures containing all components except target RNA, were included with each of the RT-PCR runs. To verify that amplified products were derived from mRNA and did not represent genomic DNA contamination, representative PCR mixtures for each gene were run in the absence of the RT enzyme after first being cycled to 95°C for 15 min. In the absence of reverse transcription, no PCR products were observed.

### siRNA Transfection

HPAECs grown to ∼50% confluence in 6-well plates or chamber slides were transfected with Gene Silencer® (Gene Therapy System, Inc. San Diego, CA, USA) transfecting agent containing scrambled siRNA (50 nM) or siRNA for target proteins (50 nM) in serum-free EBM-2 medium according to manufacturer's recommendation. To optimize conditions for efficient transfection, HPAECs were transfected with Fl-Luciferase GL2 Duplex siRNA (Target Sequence: 5′-CGTACGCGGAATACTTCGA-3′, Dharmacon, CO, USA) as a positive control. After 3 h transfection, 1 ml of fresh complete EGM-2 medium containing 10% FBS was added, cells were cultured for additional 72 h, and analyzed for mRNA level by real time PCR or protein expression by Western blotting. Scrambled siRNA control or siRNA transfected cells were subjected to scratch or wound healing experiments as described earlier.

### Lipid Extraction and Sample Preparation for LC/MS/MS

Cellular lipids were extracted by a modified Bligh and Dyer procedure with the use of 0.1N HCl for phase separation as described in [31]. C17-S1P (40 pmol) was employed as internal standard, and was added during the initial step of lipid extraction. The extracted lipids were dissolved in methanol/chloroform (4∶1, v/v), and aliquots were taken to determine the total phospholipid content as described [37]. Samples were concentrated under a stream of nitrogen, re-dissolved in methanol, transferred to auto sampler vials, and subjected to S1P-DHS1P LC/MS/MS analysis.

### Analysis of Sphingoid Base-1-Phosphates

Analyses of sphingoid base-1-phosphates were performed by electrospray ionization tandem mass spectrometry (ESI-LC/MS/MS). The instrumentation employed was an API4000 Q-trap hybrid triple quadrupole linear ion-trap mass spectrometer (Applied Biosystems, Foster City, CA, USA) equipped with a turboionspray ionization source interfaced with an automated Agilent 1100 series liquid chromatograph and autosampler (Agilent Technologies, Wilmington, DE, USA). S1P and DHS1P were analyzed as *bis*-acetylated derivatives with C17-S1P as the internal standard employing reverse-phase HPLC separation, negative ion ESI, and MRM analysis. Details of this approach are described in [38].

### Statistical Analysis

Analysis of variance and Student-Newman-Keul's test were used to compare means of two or more different treatment groups. The level of significance was set to p<0.05 unless otherwise stated. Results are expressed as mean ± S.E. M.
